# Towards an integrated surveillance of zoonotic diseases in Burkina Faso: the case of anthrax

**DOI:** 10.1186/s12889-022-13878-3

**Published:** 2022-08-12

**Authors:** Sougrenoma Désiré Nana, Jean-Hugues Caffin, Raphaël Duboz, Nicolas Antoine-Moussiaux, Aurélie Binot, Potiandi Serge Diagbouga, Pascal Hendrikx, Marion Bordier

**Affiliations:** 1 ASTRE, University of Montpellier, CIRAD, INRAE, Montpellier, France; 2 CIRAD, UMR ASTRE, Montpellier, France; 3 West African Polytechnic University, Dakar, Senegal; 4 CIRAD, UMR ASTRE, Dakar, Senegal; 5 IRD, Sorbonne University, UMMISCO, Bondy, France; 6 National Laboratory for Livestock and Veterinary Research, Senegalese Institute of Research in Agriculture, Dakar, Senegal; 7 Fundamental and Applied Research for Animals and Health Research Unit, Faculty of Veterinary Medicine, University of Liege, Liege, Belgium; 8 Research Institute of Health Sciences, Ouagadougou, Burkina Faso; 9 Etudes Formation Et Recherches Développement en Santé, Ouagadougou, Burkina Faso; 10 High Council for Food, Agriculture and Rural Areas, Paris, France

**Keywords:** Anthrax, Integrated, Governance, One Health, Surveillance, Zoonoses

## Abstract

**Background:**

Anthrax is a zoonotic disease that causes frequent outbreaks in livestock and fatal human cases in Burkina Faso. Effective surveillance of this disease calls for the establishment of an integrated surveillance system, in line with the One Health concept. However, despite a strong technical and financial support from international partners, surveillance is still poorly conducted within an integrated approach. Based on stakeholder perspectives, the study has for objective to deepen our understanding of the anthrax surveillance system and to identify the obstacles and levers towards a more integrated approach to anthrax surveillance in Burkina Faso.

**Methods:**

The data was collected from a literature review and interviews with surveillance stakeholders. We first conducted a qualitative descriptive analysis of the data to characterize the surveillance system (programmes, actors, collaboration). In a second step, we conducted a thematic analysis of the informants' discourse in order to identify what represents an obstacle or, conversely, a lever for a more integrated approach to anthrax surveillance.

**Results:**

The surveillance system of anthrax in Burkina Faso includes three programmes (in the livestock, wildlife and human sectors), which involves 30 actors. These sectoral programmes operate almost independently from one another, although some collaborations are existing for the governance and implementation of surveillance activities. Analysis of the discourse of key stakeholders led to the identification of four categories of factors that may influence the implementation of an integrated surveillance system in the country: knowledge; technical, organizational and social capacities; motivation; intersectoral governance.

**Conclusions:**

This study highlights the difficulty of translating One Health governance to the national level and the need to better articulate the visions of all categories of stakeholders. This study also reveals the need to develop specific evaluation systems for integrated policies in order to provide credible evidence of their added value for a better management of zoonotic diseases. Finally, our study underlines the need to act upstream the emergence of zoonoses and allocate more resources to the prevention of zoonoses than to their control.

**Supplementary Information:**

The online version contains supplementary material available at 10.1186/s12889-022-13878-3.

## Background

Burkina Faso is a landlocked country in West Africa, which experiments regular outbreaks of anthrax mainly in the south-west and Sahel regions [1]. Anthrax is a zoonotic disease^1^ caused by an aerobic spore-forming bacterium, *Bacillus anthracis*. The disease primarily affects herbivorous animals and can be accidentally transmitted to humans [2]. In the form of spores, the bacteria survives in the environment for decades before infecting a new host [3]. From 2009 to 2021, a total of 101 outbreaks were reported that have resulted in 535 sick animals among which 361 were fatal. The human cases associated with each of these livestock outbreaks have varied in number, but have been mostly fatal. In 2017, the country has recorded 15 cases including 5 deaths and in 2021 4 cases including 2 deaths [4]. However, both human and animal cases are likely to be underestimated.

Because of its strong impact on the health of livestock and humans, as well as on the household economy, the disease has been ranked first in the list of priority diseases jointly established by the ministries in charge of human health, animal health and the environment of Burkina Faso [4].

Epidemiological surveillance is based on the systematic and continuous collection of data to monitor the health status and risk factors of a defined population with the objective of compiling and analysing them, and then to disseminate timely information that contributes to the planning, implementation and evaluation of risk-management measures [5]. Epidemiological surveillance of zoonotic diseases requires the establishment of integrated surveillance systems that bring together the surveillance programmes operating in the human, animal and environmental sectors in order to improve the information produced and its use for better health management [6]. Indeed, intersectoral collaboration for the governance and implementation of surveillance activities, including integration of data and sharing of information on animal, human and environmental health, is increasingly seen as key to efficient health systems [7, 8]. This is in line with the One Health concept defined by the Joint Tripartite of the Food and Agriculture Organization (FAO), the Word Organisation for Animal Health (OIE), and the World Health Organization (WHO), along with the United Nations Environment Programme (UNEP), as an integrated, unifying approach that aims to sustainably balance and optimize the health of people, animals and ecosystems.^2^ The concept recognizes that the health of humans, domestic, and wild animals, plants and the wider environment (including ecosystems) are closely linked and interdependent. It requires the mobilization of multiple sectors, disciplines and communities at varying levels of society to work together. These organisations have committed to collaborate and coordinate their efforts in response to global health risks at the human-animal-ecosystem interface [9], including in the field of early-warning and surveillance systems for zoonotic diseases [10]. Among other things, they have jointly developed a guide to support countries in their fight against zoonoses using a One Health approach [11].

**Fig. 1:**
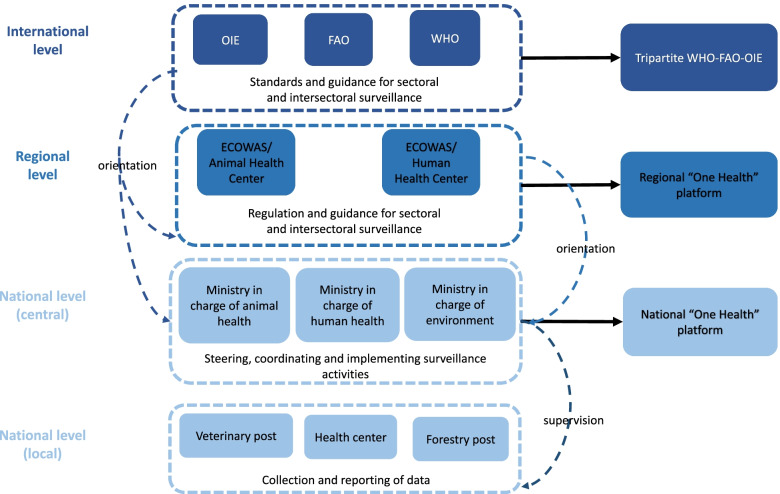
The governance framework of surveillance for zoonotic diseases in Burkina Faso

In Burkina Faso, zoonoses surveillance, including anthrax, is an official mission carried out by health authorities, namely the Ministry of Health (MOH), the Ministry of Animal Resources and Fisheries (MARF), and the Ministry of the Environment, Green Economy and Climate Change (MEGECC). In order to ensure the synergy and complementarity of their actions for the prevention and management of threats to public health, these ministries have joined forces in a One Health Platform set up in November 2019 under the direct responsibility of the prime minister and with the strong support of technical and financial partners (TFPs).^3^ This integration effort is part of a regional dynamic. At the regional level, there is a collaboration on a regional platform between the organizations of the Economic Community of West African States (ECOWAS) in charge of animal health (the Regional Animal Health Centre, or RAHC) and of human health (the West African Health Organization, or WAHO). This platform, which is still in its development phase and aims to promote and harmonize the integrated management of health issues within ECOWAS member countries. Institutional arrangements in place to govern zoonotic surveillance at the international, regional and national levels are presented in Fig. 1.

However, to date, in Burkina Faso, surveillance activities of zoonotic diseases are still very poorly governed and for the most part not conducted within an integrated approach. Using the example of anthrax, this study explores how key surveillance stakeholders understand and position themselves in relation to the new intersectoral health governance mechanisms established with the One Health Platform and to collaboration for surveillance of zoonotic diseases. Based on stakeholder perspectives, the study has for objective to deepen our understanding of the anthrax surveillance system and its implementation context, and to identify the obstacles and levers towards a more integrated approach to anthrax surveillance in Burkina Faso.

## Methods

### Data collection

The data were collected between March and October 2021 from two sources: a literature review and interviews with surveillance stakeholders.

The aim of the literature review was to: (i) analyse the institutional context and the scientific and technical framework in which anthrax surveillance is carried out at the international, regional and national (central and local) levels; (ii) describe the organization and functioning of the surveillance programmes in place, including the roles and missions of the different categories of actor; and (iii) identify the existing collaboration between these different programmes. The documents were selected by consulting the websites of the various bodies involved in the epidemiological surveillance of zoonoses at international (OIE, FAO, and WHO) and regional levels (RAHC and WAHO) or by contacting them directly. In total, we retrieved 38 documents from international (*n* = 11), regional (*n* = 8) and national (*n* = 19) organizations. These were standards (*n* = 6), regulations (*n* = 9) and guides (*n* = 23). They concerned the human health (*n* = 11), animal health (*n *= 9) and environmental (*n* = 4) sectors, or implied all sectors (*n* = 14).

In parallel, we conducted semi-structured interviews with representatives of the different categories of stakeholder involved in the surveillance. We started with a first round of interviews of stakeholders identified during the literature review. We followed up with interviews of stakeholders identified during the first round. In total, 36 people were identified and invited by email or telephone to participate in an interview. An information note on the study accompanied the email invitation or was read out during the phone call. All interviews were conducted following the same guide, and consisted of five parts: the informant's professional background and description of surveillance activities; their interactions with other surveillance actors working in the same surveillance programme or in a different one; the improvements they envision for more efficient surveillance; influence of the context (health crises, global health governance) on their collaboration in surveillance activities; and their personal expectations with regard to integrated anthrax surveillance. In total, we conducted 29 interviews with 36 informants (some were conducted with more than one) representing the different categories of surveillance stakeholders, namely: national health authorities (six interviews with six informants at central level and height interviews with 12 informants at local level), diagnostic laboratories (three interviews with five informants), professional organizations (three interviews with three informants), health practitioners (two interviews with two informants) and TFPs (four interviews with five informants at the international level and three interviews with three informants at the regional level). These informants were involved in either the human health (10 informants), animal health (18 informants), or environment (4 informants) sectors, or were operating across several sectors (4 informants). The number of interviewees for each interview varied between one to three. The interviews lasted between 60 and 90 min. All interviews were conducted in French and recorded. They were then manually transcribed. The interviews also allowed for new documents to be obtained or identified, further enriching the literature review.

### Data analysis

We first conducted a qualitative descriptive analysis of the data collected from literature review and interviews to characterize: (i) the organization of the anthrax surveillance system in Burkina Faso in terms of the programmes included and the actors involved; and (ii) the collaboration in place between the different sectors. The characteristics of these programmes and actors were identified against a list of organizational and functional attributes identified from similar studies [12] or inductively identified during the study (Table 1 and Supplementary file 1). Collaboration was identified and analysed at both governance and operational level, following the conceptual framework defined by Bordier et al., 2019 [13].

**Table 1:** Characteristics of the surveillance programmes for anthrax in Burkina Faso

Name	Owner	Sector	Domain	Regulatory status	Geographical coverage	Surveillance strategy	Hazards under surveillance	Population	Objective	Purpose
RESUREP	MARF	Animal health	Public	Official	National	Event-based	Animal diseases (including zoonotic diseases)	Livestock	Early detection, Trend monitoring	Rapid risk management, Improving knowledge
SSEF	MOH	Human health	Public	Official	National	Event-based	Human diseases (including zoonotic diseases)	Human population	Early detection, Trend monitoring	Rapid risk management, Improving knowledge
SSEI	MEGECC	Environment	Public	Official	National	Event-based	Zoonotic diseases	Wildlife	Early detection, Trend monitoring	Rapid risk management, Improving knowledge

*MEGECC* Ministry of the Environment, Green Economy and Climate Change, *MOH* Ministry of Health, *RESUREP* Epidemiological Surveillance Network for Animal Diseases, *MARF* Ministry of Animal Resources and Fisheries, *SSEF* wildlife event monitoring system, *SSEI* integrated epidemiological surveillance system

In a second step, we conducted a thematic analysis of the informants' discourse in order to identify what represents an obstacle or, conversely, a lever for a more integrated approach to anthrax surveillance in Burkina Faso. Thematic analysis is a method used in qualitative research to identify, analyse and report on themes and trends in qualitative data [14]. To apply this method, we followed the four steps described by Yin (2011) [15]: compilation, disassembly, reassembly and interpretation of the data. First, we transcribed all interview recordings and coded them according to the informant's sector, occupation and level of action. Then, using the computer-assisted qualitative data analysis software Atlas.ti, we identified in the informants' narratives words or phrases that could constitute an influential factor in the implementation of an integrated surveillance system for zoonoses in Burkina Faso, which we sorted according to the themes, concepts or ideas to which they referred. This coding was done inductively and iteratively. During the analysis of the last interviews, no new codes emerged. This suggests that we reached data saturation. The various codes were finally grouped into coherent themes describing particular levers for or obstacles to integrated surveillance. The thematic analysis was carried out independently by two researchers, a Burkinabe doctoral student in epidemiology who was familiar with the context of the study, and a researcher on integrated approaches to health who was not familiar with the field. The results obtained (themes, codes and associated quotations) were compared. Where differences in terminology were identified, the researchers agreed on a common term. Where differences in interpretation of the raw data were identified, each researcher justified his or her choice in order to reach a consensus.

### Ethical approval and consent to participate

At the beginning of each interview, written informed consent was sought from the interviewees after they were reminded of detailed information about the study, the purpose of the interview and its estimated duration. The anonymity of the informants and the confidentiality of the data were respected throughout the study. This study was evaluated and validated by the ethics committee for research in health of the Ministry of Higher Education, Scientific Research and Innovation of Burkina Faso in March 2021 (deliberation n 2021–07-161).

## Results

### Surveillance of anthrax in Burkina Faso

#### Organization and functioning of the anthrax surveillance system in Burkina Faso

Surveillance of anthrax in Burkina Faso includes three surveillance programmes, the characteristics of which are presented in Table 1.

In livestock, surveillance is event-based (i.e. passive) and coordinated by the general directorate of the veterinary services (DGSV) of the MARF through the epidemiological surveillance network for animal diseases (called RESUREP). Data collection and sampling are carried out by the veterinary posts under the DGSV. They are assisted in this task by the volunteer extension workers at the village level, who are under their direct responsibility, and the heads of the zones for technical support to livestock, who are under the hierarchical responsibility of the provincial and regional directorates of the MARF. The samples are sent to the regional livestock laboratories, which are responsible for packaging and shipping them to the national livestock laboratory, which is in charge of testing them. The heads of the veterinary posts notify suspected cases on a weekly basis to the epidemiology department of the DGSV's Animal Health Directorate, which centralizes all surveillance data. The data are analysed weekly and the results recorded in epidemiological bulletins, which are sent to the DGSV, the TFPs and also to the heads of the veterinary posts. Notification of positive cases is made to the OIE and WAHO.

In the human sector, there is an event-based surveillance programme supervised by the MOH. This surveillance is coordinated by the national committee in charge of outbreak management, whose executive body is the epidemiological surveillance service of the directorate in charge of population health protection (DPSP). The data are collected and reported by the health facilities consisting of the centres in charge of health and social promotion, the regional hospitals, the university hospitals and the hospitals. Additionally, there is a network of community-based health workers who report health events to the centres in charge of health and social promotion. Each week, the health facilities summarize the diseases and events under surveillance in the weekly official letter telegram notebooks. The summary of surveillance data is transmitted by telephone to the district level, then from the district to the regional level and finally from the region to the DPSP, which processes and analyses the surveillance data. The results are transmitted to the WHO country office and WAHO, using an electronic-based reporting tool called District Health Information System 2.0 (DHIS2).

There is no environmental surveillance as such. In the event of unusual wildlife mortality, forestry officers alert the heads of veterinary posts, who take charge of surveillance according to the organization of RESUREP. Notification is then made to the provincial and regional directorates in charge of the environment. Forestry officers are assisted by eco-guards in detecting and reporting wildlife health events.

Overall, the information produced by the surveillance system is used to trigger rapid risk management, but the data are not analysed to monitor long-term trends nor used to guide public policy.

**Fig. 2:**
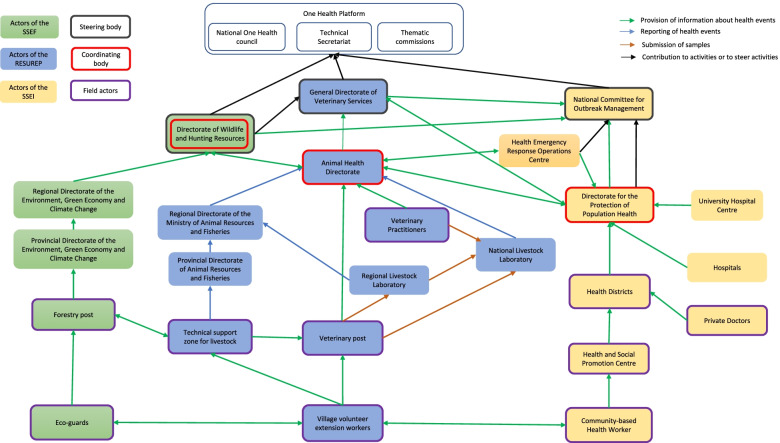
Actors of the surveillance system for anthrax in Burkina Faso: role and interactions

In total, the surveillance system involves 30 actors from the following categories: central (11) and local (8) health authorities, private practitioners (2), health facilities (4), laboratories (2), and community workers (3). Their characteristics are presented in Fig. 2 and in supplementary files.

## Collaboration between sectoral surveillance programmes

**Fig. 3:**
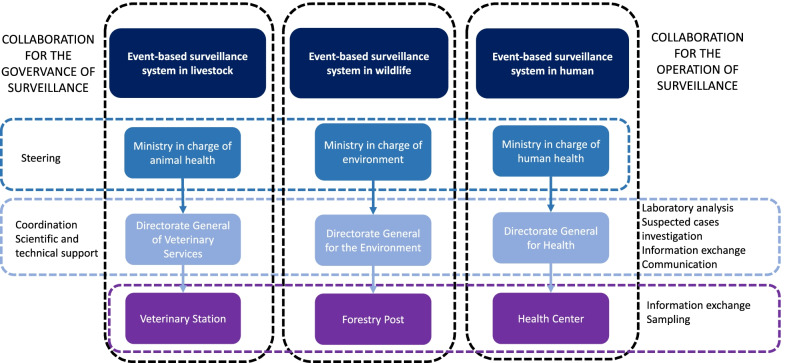
Collaboration between sectoral surveillance programmes for anthrax in Burkina Faso

The sectoral surveillance programmes operate almost independently from one another. However, a number of collaborative modalities have been identified and described for both the governance and the implementation of surveillance activities (Fig. 3).

The One Health Platform, through its tasks and constitution formalized in its creation and operation decrees, can be seen as the body responsible for the governance of integrated surveillance of zoonotic diseases, including anthrax. Within the One Health Platform, the national One Health Council, comprising the ministers and heads of the technical general directorates, is responsible for establishing the national roadmap for integrated health risk management and ensuring synergy of action between sectors. The Council is intended to meet at least once a year. The technical committee, which is the executive body of the Council, is supposed to monitor the implementation of the roadmap and ensure the proper functioning of the thematic commissions, through meetings held at least twice a year. Meetings of these two bodies had not yet been held at the time of the study. Coordination is managed by the Technical Secretariat, housed in the MOH and composed of a permanent secretary and sectoral experts currently being appointed by their respective ministries. It prepares the administrative and regulatory acts necessary for the functioning of the One Health Platform, and monitors the implementation of the directives, decisions and recommendations of the Council and the Committee. It brings together the focal points of each ministry and the TFPs once a week to share information on ongoing activities. As the steering and coordination bodies are not yet fully functional, the role of the Secretariat is still limited. The One Health Platform is also made up of seven multisectoral thematic groups that provide scientific and technical support to the different bodies. The degree of functionality varies from one group to another, and those that could be more specific to integrated anthrax surveillance (i.e., zoonoses and surveillance groups) are not yet fully established.

With regard to the implementation of surveillance activities, collaboration exists at several stages of the surveillance process. The animal health and wildlife surveillance programmes are particularly connected. Indeed, once a suspected case has been established by the environmental officers, the veterinary officers and their partners take over the rest of the process, as described for the operation of the RESUREP. Forestry officers are currently being trained by the veterinary services to take samples themselves. Collaboration also exists between the animal-health and human-health sectors. Transfers of skills and the sharing of technical equipment and reagents can occur on an ad hoc basis. There are also several initiatives led by TFPs to harmonize the packaging and routing of samples to the laboratories and to establish a common information system for surveillance results using DHIS2. Surveillance data on priority zoonoses collected at pilot sites were centralized until 2020 in a common database, set up by the Measure Evaluation project [15]. However, those initiatives have been struggling to keep up their maintenance with domestic funding, since external sources of fund have stopped. In the event of a suspected case of anthrax in one sector or another, joint investigations between the animal and human health authorities are conducted. The sectors exchange their respective surveillance results during the quarterly meetings of the national centre in charge of outbreak management. Although not formalized, collaboration between the sectors seems to exist in a more routine and fluid manner at the field level. Local actors systematically exchange information on health events brought to their attention, and jointly conduct awareness-raising activities for the population. In the event of suspected cases, they coordinate to take the first management measures before the intervention of the central authorities' multisectoral teams.

### Levers for and obstacles to the implementation of a more integrated approach to surveillance of anthrax in Burkina Faso

**Fig. 4:**
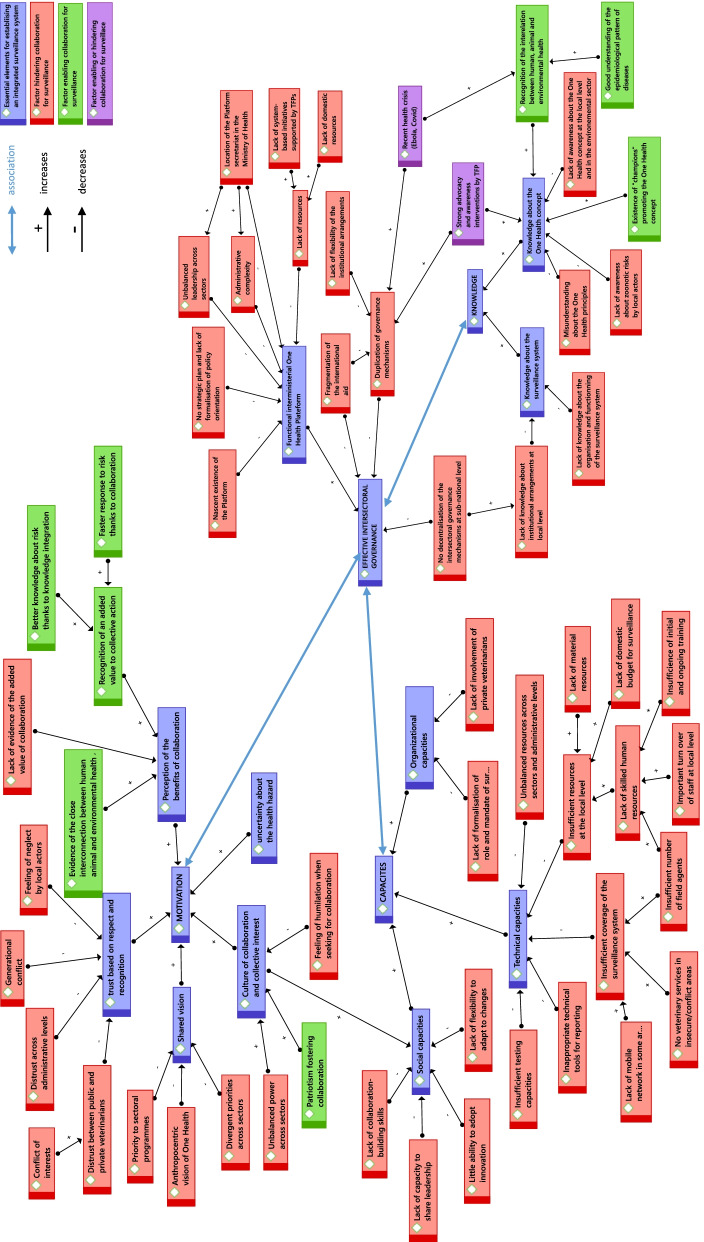
Obstacles to and levers for an integrated surveillance system of anthrax in Burkina Faso

Analysis of the discourse of representatives of key stakeholders in anthrax surveillance in Burkina Faso has enabled us to identify factors that may influence the implementation of an integrated surveillance system in the country. These factors fall under four general themes (Fig. 4). The first three are directly related to the intrinsic qualities of actors to engage in collaboration: their knowledge; their technical, organizational and social capacities; and their motivation. The fourth theme relates to the governance of intersectoral surveillance.

#### The level of knowledge of surveillance actors

Integrated surveillance calls for collaboration between institutional and civil-society actors operating in different sectors to optimize surveillance [6]. Knowledge of the foundations of the One Health concept and of the institutional arrangements put in place to support its operationalization is therefore a prerequisite for stakeholder engagement in collaboration.

In Burkina Faso, the health authorities at the central level have appropriated the One Health concept and are aware of the importance of collaboration and multi-partner approaches to managing complex health problems, as reflected in the discourse of informant A1: *"If the [One Health] concept did not exist it would need to be created because it allows health problems to be managed jointly, it really allows health problems to be managed in a short time and with few resources, and in any case we achieve better results than leaving each one to fend for itself.”* This high level of awareness is the result of two major factors: the strong efforts made by the TFPs to raise awareness of the approach, and the latest health crises in the region (COVID-19, Ebola), which have served as proof of the value of the approach. This was mentioned by informant A22 on the subject of Ebola: *"There is a disease in the region that has changed the situation and shown us that animals are a major source of disease."* Awareness was then reinforced at national level by the existence of “*pioneers, emerging researchers*” (A22) who actively advocated for the concept. However, the appropriation of the concept varies from one sector to another, with a lower level in the environment sector, which has benefited less from the support of the TFPs. It also varies from one decision-making level to another, with the local level considered to be less well aware than the central level. Actors at the central level also emphasize the need to strengthen zoonotic risk awareness activities at the level of the deconcentrated services and in the communities to reduce the risk of contamination and encourage the notification of suspected cases. Conversely, poor knowledge of the concept can have a deleterious effect on collaboration. Indeed, some actors understand the approach as an allocation of sectoral missions to a select few and not as a pooling of expertise through collaboration. They therefore fear that adopting the approach can lead to a loss of their prerogatives and power. Knowledge of the epidemiological cycles of diseases, which demonstrate the link between human, animal and environmental compartments, also plays a role in collaboration between the different sectors and actors. In the specific case of anthrax, it allows actors to recognize the need for concerted action to manage the risk, as intervention in one compartment alone is not sufficient to control the disease.

Interaction with others requires knowledge of the organization and of the functioning of all the surveillance programmes, as well as of the relevant institutional arrangements. This knowledge is relative and varies according to the administrative level. Surveillance actors have very limited knowledge of how surveillance networks function in other sectors, and sometimes even of their own sector and the importance of their role in the network, as indicated by informant A2's narrative: *"[W]hen the bulletins [of surveillance results] leave my office, I don't know exactly where they go."* While the central authorities are generally well informed of the existence of the One Health Platform and its functioning, this is not the case at the local level, which was not consulted when this collaborative mechanism was established and which has not been made aware of its roles and missions.

### Stakeholder capacities for integrated surveillance of anthrax

We identified different types of capacity that could impact on the implementation of an integrated surveillance system, namely technical, organizational and social.

The quality of an integrated surveillance system depends to a large extent on the performance of each of its programmes, which in turn depends on the technical skills of the actors [16]. We found the discourse of informants emphasized that sectoral surveillance in Burkina Faso has many technical shortcomings. In the animal and environmental sectors, the interviewees were unanimous in deploring the lack of adequate resources, particularly at the local level, which is the first line of detection of cases in event-based surveillance systems. There is *"no budget to accompany surveillance actions. There is no budget line at the state level,"* according to informant A1. Informant A27 pointed out that some agents finance surveillance activities from their own funds: *"Sometimes you have to give something from your own pocket to encourage him [the field agent] and then he gets up to go and take the data."* There are not enough staff to cover the whole territory effectively and the lack of vehicles and fuel reduces their range of action. *"As there is no fuel provided, it will follow that he [the field officer] will not go there [when there are suspected cases and this is lost information"* according to informant A3. Initial and ongoing training in surveillance practices is insufficient, and the high turnover of officers at local level exacerbates the lack of skill maintenance. Informant A22 ironically described the actors in surveillance as belonging to two categories: *"frustrated old people"* and *"young people with no experience."* The paper-based alert system is considered too archaic to allow for rapid and quality notification and response. In the pilot sites that were equipped with tablets for electronic notifications, field officers *“closed the tablets because they no longer had an internet connection when the [TFP-funded] project stopped,”* as informant A20 pointed out. The lack of resources was also found to affect the entire laboratory network. The regional laboratories are almost non-operational due to a lack of budget. *"Even if you want to buy a box of matches, you have to go to the Regional Directorate or take it out of your pocket,"* according to informant A8. Technical capacities are considered to be more advanced and territorial coverage more extensive in the human health sector compared to other sectors. This sector receives more material and financial resources and capacity building from the government and TFPs. This imbalance is considered by several actors to be an obstacle to collaboration at the central and local levels and *"one of the primary objectives is to bring the different ministries up to standard"* according to informant A20. This is particularly the case for the Ministry of the Environment, which currently has *"no surveillance specialists, nor any surveillance system,"* said informant A1.

This lack of technical capacity is reinforced by insufficient organizational capacity of surveillance actors. Indeed, several informants pointed to the lack of clear formalization of the roles and missions of each actor for surveillance activities, as underlined by informant A3 *"there is total confusion between actors because the service is not codified and everyone does the same thing [there is duplication of tasks]."* Moreover, private veterinarians are very little involved in the surveillance network, even though they are considered by the other actors to be key elements of health surveillance because of their range of action, their frontline position, their established position in the community and their privileged contacts with farmers. Current contextual factors also negatively influence the organizational capacities of surveillance actors. These include insecurity, which makes certain areas inaccessible for conducting surveillance activities, and the lack of telephone coverage in certain areas, which hinders the proper circulation of information.

This lack of capacity affects the quantity and quality of health data collected in the different sectors, and therefore also the exchange of relevant health information between sectors for an effective management of zoonoses.

In addition to the technical and organizational skills needed to set up an integrated surveillance system, social skills are also essential to enable actors to interact with each other [17]. The lack of soft skills among surveillance actors was pointed out by several informants. Surveillance actors are technical actors, who were described as lacking proactivity and innovation when it comes to developing collaboration and adapting to changes. Sharing leadership during investigation missions and the ability to work as a team remain difficult in the field.

### Motivation of stakeholders to engage in collaboration for surveillance

Collaboration requires significant effort on the part of the actors because it consumes resources and requires significant efforts to adapt [12]. Motivation to collaborate is therefore a key element in the implementation of an integrated surveillance system. In Burkina Faso, the motivation of stakeholders is influenced by several factors: the perception of the benefits of collaboration; the culture of collaboration and collective interest; the existence of a shared vision; trust based on respect and recognition.

The perceived benefit of collaboration is driven by two main factors. Firstly, many informants, at local, central and supranational levels, recognize an added value to collective action compared with individual action. This added value may lie in better risk management due to the integration of greater knowledge and expertise, because “*everyone is aware that there are subjects that cannot be tackled in isolation*” (A12). But above all, it relates to the improvement of the performance of surveillance and response, particularly in terms of reducing response times, which allows the problem to be *"brought under control in record time"* (A1). The benefits of collaboration are also made clear by evidence of the close interconnection between human, animal and environmental health, which require coordination interventions across sectors to ensure their efficiency. In the regional health context, stakeholders are fully aware of the role played by the animal compartment in the emergence of new zoonoses (Ebola). Countries that have experienced Ebola are described as "*more aware and more advanced in the development of [One Health] platforms*" than other countries in the region, as stated by informant A22. However, several informants recognized the need for more evidence of this added value, and better communication about it, to ensure the long-term commitment of all categories of stakeholders.

The analysis of the informants' comments also revealed the existence of a culture of collaboration and collective interest that varies between categories of stakeholders, as well as within individual categories. At the international level, the animal health sector was described as much more proactive in developing collaboration with other sectors. TFPs described collaboration to be more functional at the local level than at the central level, and this has been substantiated by external evaluations. Local actors considered that the collaborative mechanisms institutionalized with the One Health Platform essentially allow for improved collaboration between ministries at the central level, as they see the collaboration already functioning properly at the local level. This is illustrated by the words of informant A4 about the Platform: *"I don't know what they are innovating, but [collaboration they are establishing at central level with the Platform] we are already doing it on the ground"*. Analysis of the actors' discourse shows that the meaning of collaboration varies greatly from one individual to another, depending on their values and reasoning. For instance, the relations between public and private veterinarians can be either collaborative and partnership-based or highly conflictual, depending on the posture adopted by the individuals. Several informants also considered that involvement in surveillance and case reporting was a matter of patriotism and should be done automatically even in the absence of compensation. Others considered that going to another person to ask for their collaboration is a humiliation.

The existence of a shared objective is a key element in developing sustainable collaboration [17]. Although anthrax is officially recognized as a priority zoonosis by the three ministries in charge of health, their vision and priorities are not convergent. Each ministry has its own programme and roadmap with defined objectives, where little room is left for collaboration between sectors. Moreover, the health authorities in the human sector defend a very anthropocentric vision of the One Health concept, in which the purpose of collaboration is to protect human health, as described by informant A16: *"[W]hether the disease is of environmental or animal origin, [we need] to be able to work together to ensure that people, if they are affected, regain their health and well-being."* This position is shared by several TFPs. According to some informants, this anthropocentric approach is responsible for the location of the permanent secretariat of the One Health Platform within the Ministry of Health, for which zoonotic diseases are not a priority. For anthrax more specifically, informant A12 specifies that *"[a]nthrax does not carry the same weight in the activity of a health worker as the fight against polio or malaria. However, at the level of veterinary services, it is a real priority and people mobilize very quickly."* According to informant A13, this difference in priorities is reflected in the fact that, at the level of the TFPs, the animal health sector *"took the lead and oversaw the implementation of the Tripartite at the regional level."*

Collaboration is also based on trust [18], which in turn depends on recognition and respect between the actors. However, our study has highlighted a very strong feeling of not being duly recognized on the part of some actors and also of mistrust between certain categories of actor. At the sub-national level, actors feel neglected and discredited, as they have no decent resources to carry out their activities and do not benefit from the actions implemented by TFPs. As a result, *"people are gradually losing morale"* (informant A27), *"they are frustrated"* (informant A4) and *"sometimes they rebel"* (informant A15). There is also a conflict between the younger and older generations. While the older generation sees the younger generation as greedy, materialistic, selfish and incompetent, the younger generation sees the occupation of certain positions by older people as hindering the adoption of innovations for better zoonotic disease surveillance and other change. There is also a strong sense of mistrust between private and public veterinarians. Private veterinarians blame the public sector for coming to provide paid private services in their area and competing with them. One private practitioner (A3) said: *“We don't have a problem with the pharmacists, our biggest competition is with the public vets”* In return, public veterinarians criticize the private sector for wanting to take over their official missions and for coming to "*spy*" on them. Finally, there is a lack of mutual recognition between the different levels of governance. The central level considers that the local level is not sufficiently involved in surveillance missions, while the local level criticizes the central level for a lack of consideration for their work, for not taking into account the problems encountered in the field and for the absence of feedback on the information they provide.

### Governance of intersectoral surveillance

Governance emerged as a key issue in the stakeholders' discourse. Overall, stakeholders deplore the fact that governance of surveillance is exercised in too top-down a manner, and that decisions taken at central level are made without consulting local stakeholders and are therefore not adapted to the realities in the field.

The governance of integrated surveillance is covered by the missions of the One Health Platform, which was implemented under the impetus of the TFPs and with external funding. The actors said they recognize that the implementation of this platform has improved collaboration between the different sectors, either by formalizing what already existed or by creating a framework for consultation that is conducive to new interactions. However, they underlined the need for major formalization efforts to facilitate smooth collaboration and improve information sharing, through the issuing of a strategic plan at ministerial level and through the development of protocols at the operational level. Moreover, several categories of actors do not consider the Platform functional or operational fully enough to meet its objectives or to engage all actors. First of all, even though particular attention has been paid to ensuring that the chairmanship positions of the various bodies is distributed among the different sectors and institutions, the permanent secretariat is placed at the level of the Ministry of Health. For some actors, this results in an orientation towards priorities that do not necessarily meet the expectations of other sectors, which can have a negative impact on their commitment. This organization also delays the ratification of decisions because they have to go through a lengthy inter-ministerial validation process. In Burkina Faso, the health sector is described by actors as much more powerful than the animal health or environment sectors, particularly in terms of human resources and capacity to mobilize funding to meet its sectoral priorities. There is therefore an imbalance being created in terms of leadership to govern health issues. Secondly, the One Health Platform sorely lacks adequate resources to function, particularly for its steering and coordination bodies. There is still no specific domestic budget dedicated to the Platform's activities, to train staff from the various ministries in the approach or to enable collaborative activities in the field (e.g. joint case investigations). The staff of the permanent secretariat is made available by the different ministries. The experts are completely discharged from their sectoral missions, but the permanent secretary only allocates 20 per cent of his time to managing the platform. Finally, for intersectoral governance to be fully functional, the approach must be disseminated within the various institutions in the central and decentralized services. For the time being, several actors, at both central and local levels, considered the One Health Platform a outside body because decisions taken at ministerial level do not direct the roadmaps of the institutions towards a more integrated approach. Moreover, the Platform was intended to be deployed in each region, under the aegis of the governors, but it has been slow to take effect, in particular because governors are not convinced of this need and see One Health "*as science fiction*" (informant A20). According to the informants (A2, A4, A9, A14, A30), this regional deployment is nevertheless essential to allow local actors to better appropriate the new collaborative mechanisms, with which they acknowledge not being sufficiently familiar, due to lack of information.

Several actors lamented the fact that the One Health Platform duplicates other institutional arrangements already in place, such as the NCOM, which could have fulfilled the functions assigned to the Platform if given the opportunity to duly review their roles and missions and include new partners. This is partly due to the fact that "*the concept is fashionable*" and that each country wants its own branded One Heath Platform rather than recycling existing mechanisms that are not clearly labelled "One Health." This contributes to a dispersion of already limited resources. Other stakeholders see the platform as an opportunity to increase the visibility of their sector, which is not well represented in the current mechanisms, and as a megaphone to advocate for the allocation of more resources to the management of zoonoses.

The platform is very recent and the actors said they believe that it will become more functional over time. “*It is like when you bring a child into the world, it cannot walk at the same time, it has to grow up, it has to get vaccinations, it has to go on all fours, it has to learn to stand up and then it has to walk”* (informant A22).

Intersectoral governance in Burkina Faso is significantly influenced by all the TFPs, whether intergovernmental or international non-governmental organizations, or national cooperation agencies from third countries. Within the framework of the Global Health Agenda, many programmes have contributed to raising awareness of the importance of the One Health concept at ministerial level and also at local level. This rising recognition of the concept has resulted in a strong political commitment. The 2017 evaluation of Burkina Faso for compliance with the International Health Regulations (IHR) showed certain shortcomings in collaboration for intersectoral surveillance, and the government is keen to remedy these shortcomings as best it can by the next evaluation in 2022. However, it must be noted that the majority of resources for collaboration have been made available by the TFP rather than the national or local governments s, as underlined by informant A22: *"Today, One Health is 95 per cent financed by the World Bank, USAID [United States Agency for International Development] and other bilateral donors."* The setting up of the Platform was entirely financed by donors, and the structure of the thematic groups is modelled on the technical areas of the IHR. The prioritization of zoonoses to be monitored was technically and financially supported by the United States Agency for International Development (USAID). The TFPs more frequently tend to finance vertical programmes that are dedicated to a specific disease, particularly in the human sector. For instance, the Global Fund was essentially dedicated, before the appearance of COVID-19, to the three diseases AIDS, tuberculosis and malaria. When they finance intersectoral programmes, they prefer to finance activities of the technical groups of the Platform rather than the functioning of its steering and coordination mechanisms. Indeed, the activities of the thematic groups are more in line with the strategic plan of the TFPs, and it is easier for them to evaluate their financial and technical execution. Collaboration is therefore organized on a project-by-project basis without any precise linkage to an overall strategic plan, and local actors find it difficult to appropriate the tools and mechanisms put in place by the donors. Some actors explain this by pointing to the novelty of the proposed tools, which imply changes in practices that must be supported over the long term but are not. Others explain it by the fact that technical support is fragmented and that it responds first and foremost to donors' roadmaps before meeting the country's needs.

## Discussion

Our study has provided a detailed description of the surveillance of anthrax in Burkina Faso in relation to the regional and international contexts. The surveillance system includes three programmes, respectively, in the animal, human and environmental health sectors. Despite the establishment of new collaborative mechanisms and the influence of the TFPs, there is very little collaboration between these programmes in a One Health spirit. Collaboration is formalized at the central level via an inter-ministerial platform and its various bodies, but is not yet completely functional. At the local level, collaboration is not formalized but is effective between field agents in terms of information exchange and joint investigations.

This study has also identified 57 factors that represent obstacles to or levers for a more integrated approach to anthrax surveillance in Burkina Faso. They fall into four main categories: knowledge; capacity of actors; their motivation; and intersectoral governance. However, these categories are not compartmentalized and links exist between their constituent factors (Fig. 4). Intersectoral governance appears to be the element that structures all the factors. Indeed, the quality of governance conditions the capacity and knowledge levels of the system’s actors, levels, which then have a retroactive effect on the proper functioning of governance mechanisms. Similarly, governance affects the motivation of actors to invest and commit to collaboration, which in turn contributes to the quality of governance.

This study applies qualitative methods, which have recently gained popularity across an increasingly wide range of domains, particularly in public health and international research and development [19]. The major advantage of qualitative research lies in the richness of the data produced, which is specific to the context of implementation. It allows for the exploration of actors' beliefs, values and motivations and sheds light on certain behaviours [20]. This approach was therefore well suited to the objective of our study, as it allowed us to explore the perception and posture of the different categories of actor towards integrated surveillance from a One Health perspective. It led to a deeper and richer understanding of the technical, cultural and socio-political factors that hamper the operationalization of integrated surveillance. Interviews were conducted with all categories of actor involved in surveillance, whether they act directly in the surveillance process or have a technical and financial support role. We have also endeavoured to work at both sectoral and intersectoral levels, as well as at all levels of intervention, from local to international. By the last of our interviews, we did not obtain any additional information from the new informants, which suggested that we had reached a good level of saturation. However, while we interviewed at least one representative from each key category of actor in anthrax surveillance in Burkina Faso, we cannot certify that the discourse of any given informant was a typical representation of the views of that actor (e.g. the organization, institution, profession) as a whole. At the local level, we interviewed actors working in areas with or without previous anthrax outbreaks to capture the influence of diverse epidemiological context on the informants’ discourse.

In Burkina Faso, intersectoral health governance appears fragmented and still unable to lead actors towards a shared vision or a definition of strategic priorities that are feasible and collectively validated for integrated zoonotic disease surveillance. However, as for any other global health movement, only strong governance with coherent provisions would make it possible to operationalize the One Health concept in the field of epidemiological surveillance of zoonotic diseases [21]. This seems to be due to several factors: the absence of strong leadership at the international level to support the development of intersectoral policies; the lack of alignment between the agenda of the TFPs and that of the governments of the countries in which they operate; the priority given to curative health care at the expense of prevention; and the promotion of public management tools that are not adapted to intersectoral policies.

The recent intersectoral governance mechanisms implemented in Burkina Faso were developed under the impetus and with the financing of various TFPs. Among these TFPs, the WHO, OIE and FAO, which are the most influential organizations in guiding and governing the One Health movement, are the implementation leads of numerous initiatives at national and regional levels. However, these organizations are criticized for having adopted an unrealistic and undefined vision of the concept, without strong institutional anchoring, expressed essentially through principles and declarations that were decided during meetings and endorsed by international organisations and governments [22]. This unclear definition and lack of strong leadership has left the field open for different categories of actor to direct the operationalization of the concept to serve their own interests and has resulted in a proliferation of sometimes competing initiatives conducted under the One Health umbrella [23]. Although the Tripartite has made recent efforts to provide countries with guidelines for operationalizing surveillance in a One Health approach, such as the ones for zoonotic diseases [10], there is still a significant need for support in implementing integrated surveillance, facilitating the coordination of activities and developing collaboration-building skills among stakeholders [21].

This has resulted in the orientation of policy choices and financial allocations by TFPs towards issues that they manage or that they consider to be priorities, but which are not necessarily consistent with the needs and expectations of recipient countries [21, 24–26]. This is best illustrated by the example of Highly Pathogenic Avian Influenza (HPAI), for which considerable funds were allocated to strengthen surveillance in developing countries. This was justified mainly by the pandemic nature of the disease, which threatened developed countries, rather than its health and economic importance in developing countries, whose health authorities are much more concerned with endemic zoonotic diseases such as Brucellosis [23]. Vertical programmes have always been predominant in TFP strategies, with the majority of initiatives to strengthen collaboration between sectors focusing on specific hazards such as pandemic influenza preparedness and antimicrobial resistance [23]. Recently, TFP support is taking a new turn with more system-based initiatives such as the establishment of One Health inter-ministerial platforms deployed in Central and West African countries with USAID funding. However, these One Health platforms look like travelling models described by de Sardan [27, 28], which are established simultaneously in different countries on the assumption that they are effective and efficient by nature. However, as with any public management tool, their proper functioning depends mainly on the context and the way in which the actors use them. This need for contextualization is even more important in the case of intersectoral governance, which calls for the design of institutional arrangements that mobilize actors with different cultures and varied expectations, sometimes in competition with one another [12, 29, 30]. While it is well understood that global policies cannot be contextualized by their very nature, their lack of articulation with other scales can legitimately be criticized.

The lack of funding for integrated surveillance is very clear in the results. Currently, global health policy strategies relayed by the TFPs give priority to curative approaches to the detriment of preventive approaches, in which surveillance is involved [31–33]. For example, the objective of universal health coverage, advocated by the WHO to finance health systems, is mainly focussed on improving access to curative care. This has the effect of orienting national health policies towards financing the care of more and more patients, and away from strategies that help prevent disease.

The transfer of management control instruments from the private sector into public governance (the New Public Management) tends to focus public action on short-term measurable objectives, the performance levels of which can be attributed to identified actors [34, 35]. In West Africa, the implementation of programme budgets, which have been promoted by TFPs since the end of the 1990s, has become a regulatory standard for all ECOWAS countries since 2017. However, these instruments have been criticized for being unsuitable for the design and implementation of complex public policies, which often have long-term objectives, and mobilize actors whose interests are sometimes divergent [30, 36]. Indeed, this management method limits the allocation of resources, as well as the capacity to steer cross-cutting multi-sectoral policies. Sectoral ministries compete for resources in the budget cycle, which tends to limit intersectoral collaboration initiatives. The annual work plans of their various departments are designed for autonomous action focused on their annual performance. There is therefore no monitoring and evaluation system to measure the performance of intersectoral policies. This leads to a lack of robust evidence of their added value, which is considered essential to stimulate political and financial commitment to these approaches, as highlighted by the discourse of surveillance actors in Burkina Faso and in the literature [21, 37].

The intersectoral governance of zoonotic disease surveillance in Burkina Faso is therefore shaped by the strategic orientations adopted by the TFPs in the One Health movement. To date, these orientations do not seem to have led to the establishment of functional and sustainable integrated surveillance governance mechanisms at the national level, due to a lack of ownership by local actors and a lack of domestic resource allocation to ensure their functioning. Under these conditions, there is a risk that the One Health Platform will be perceived more as a receptacle for aid responding to a "fashion" of the TPFs than as a mechanism for governance of the national intersectoral health policy [26–28, 38].

## Conclusions

This study highlights the difficulty of translating One Health governance to the national level and the need to better articulate the visions of institutions at the different decision-making levels, as well as those of the community [39]. The establishment of integrated surveillance governance in Burkina Faso has been largely driven by international organizations, relying at national level on high-level political fora and neglecting the role of civil society organizations and affected communities. However, for such an integrated zoonotic disease surveillance system to be operational and sustainable, it is important that all stakeholders buy into the One Health concept [37]. Moreover, this study has allowed us to determine that stakeholders involved in anthrax surveillance have varied postures, perceptions and expectations of One Health surveillance, and of their interactions with other stakeholders in the system. This suggests that it would be necessary to co-construct with all these actors a common vision of the desired surveillance system and to collectively identify the necessary changes to move toward an integrated surveillance system that is accepted and applied. This co-construction process must be carried out within a framework that allows all parties to raise their voice freely, so that its outputs are not the result of only the most powerful actors’ input but are representative of the expectations of all parties. Different methodological frameworks, using various tools, exist to support such a co-construction of the decision-making processes, such as participatory modelling [40]. In addition, this would require the actors strengthening their collaboration-building skills at all decision-making levels to recognize and respect disciplines and professions other than their own [21].

This study also reveals the need to develop specific evaluation systems for integrated policies in order to be able to measure their effectiveness, the economic benefits, and provide credible evidence of their added value for a better management of health hazards [37, 41]. Like any other multi-stakeholder arrangement, the survival of the One Health Platform in Burkina Faso will depend on its ability to prove its added value [21]. In addition, development of standardized and robust evaluation tools for the evaluation of the impacts of integrated surveillance could generate evidence to foster the engagement of government for the allocation of necessary resources and to support the development of more precise guidelines for the governance and implementation of surveillance activities [6, 7].

Finally, our study underlines that too few resources are still dedicated to the prevention of zoonoses compared with those allocated to their control. Hence, it is time to change the paradigm and act upstream of the emergence and spread of zoonoses by setting up adapted integrated surveillance systems and, more generally, by promoting the transition towards sustainable socio-ecosystems [42]. This is notably the objective of PREZODE,^4^ an international One Health initiative supporting strategies to reduce the risk of emergence of zoonotic infectious diseases. It aims to help coordinate a large portfolio of national, regional and international projects and programmes concerning the emergence of zoonotic infectious diseases, and to implement innovative methods to improve prevention and mitigate emergence risks, in line with the recommendations of the High-Level Expert Panel on One Health of the Tripartite + (the three organizations FAO, OIE and WHO, plus UNEP).

## Supplementary Information

**Additional file 1.**

## Data Availability

The datasets generated and/or analysed during the current study are not publicly available to protect interviewees’ privacy but are available from the corresponding author on reasonable request, after anonymization of data and removal of any information that could trace back to the identity of the participant.

## Abbreviations

AIDS: Acquired immune deficiency syndrome
CDC: Centers for Disease Control and Prevention
DGSV: Direction Générale des Services Vétérinaires (general directorate of veterinary services)
DHIS2: District Health Information System, Version 2
DPSP: Direction de la Protection de la Santé des Populations (directorate in charge of population health protection)
ECOWAS: Economic Community of West African States
FAO: Food and Agriculture Organization
IHR: International Health Regulations
MARF: Ministry of Animal Resources and Fisheries
MEGECC: Ministry of the Environment, Green Economy and Climate Change
MOH: Ministry of Health
OIE: Word Organisation for Animal Health
RAHC: Regional Animal Health Centre
RESUREP: Réseau de Surveillance Epidémiologique des maladies animales (epidemiological surveillance network for animal diseases)
SSEF: Système de Surveillance Epidémiologique de la Faune (wildlife surveillance system)
SSEI: Système de Surveillance Epidémiologique Intégré (Integrated epidemiological surveillance system)
TFP: Technical and financial partners
UNEP: United Nations Environment Programme
USAID: United States Agency for International development
WAHO: West African Health Organization
WHO: World Health Organization

## Footnotes

1 https://www.who.int/fr/news-room/fact-sheets/detail/zoonoses

2 https://www.who.int/news/item/01-12-2021-tripartite-and-unep-support-ohhlep-s-definition-of-one-health

3 Decrees 2019–1086/PRES/PM/MRAH/MINEFID/MESRSI/MS/MAAH/MEEVCC and 2020–210/MS/MINEFID/MESRSI/MAAH/MRAH/MEEVCC.

4 https://prezode.org/The-initiative
