# Supplementary material for: Towards an integrated surveillance of zoonotic diseases in Burkina Faso: the case of anthrax
Source: BMC Public Health. 2022 Aug 12;22:1535. doi: 10.1186/s12889-022-13878-3 (PMC9372945; doi:10.1186/s12889-022-13878-3)
Supplement: Supplementary file 1 — Additional file 1. [file 12889_2022_13878_MOESM1_ESM.docx]

Supplementary file 1. Actors involved in the surveillance of anthrax in Burkina Faso

| Name | Activity domain | Activity sector | Profession | Discipline | Scale of activity | Supervisory authority | Surveillance programme | Role in surveillance |
| --- | --- | --- | --- | --- | --- | --- | --- | --- |
| DGSV | Public | Animal health | Health authority | Veterinary public health | Central | MARF | RESUREP | Steering |
| DSA | Public | Animal health | Health authority | Veterinary public health | Central | MARF | RESUREP | Coordination  Investigation of suspected cases |
| PV | Public | Animal health | Health authority | Veterinary public health | Local | MARF | RESUREP | Data collection  Investigation of suspected cases |
| DRRAH | Public | Animal health | Health authority | Veterinary public health | Local | MARF | RESUREP | Coordination |
| DPRAH | Public | Animal health | Health authority | Veterinary public health | Local | MARF | RESUREP | Coordination |
| ZATE | Public | Animal health | Health authority | Veterinary public health | Local | MARF | RESUREP | Data collection |
| VVV | Public | Animal health | Community Agent | Veterinary public health | Local | MARF | RESUREP | Data collection |
| Veterinary Practitioners | Private | Animal health | Private practitioners | Veterinary medicine | Local | MARF | RESUREP | Data collection |
| LNE | Public | Animal health | Laboratory | Microbiology | Central | MARF | RESUREP | Laboratory testing |
| LRE | Public | Animal health | Laboratory | Microbiology | Local | MARF | RESUREP | Sending the samples  Investigation of suspected cases |
| DFRC | Public | Environment | Health authority | Ecology | Central | MEGECC | WSS | Steering,  Investigation of suspected cases |
| DPEEVCC | Public | Environment | Health authority | Ecology | Local | MEGECC | WSS | Coordination |
| DREEVCC | Public | Environment | Health authority | Ecology | Local | MEGECC | WSS | Coordination |
| PF | Public | Environment | Health authority | Ecology | Local | MEGECC | WSS | Data collection  Investigation of suspected cases |
| Eco-guards | Public | Environment | Community agent | Ecology | Local | MEGECC | WSS | Data collection |
| CNGE | Public | Human health | Health authority | Public health | Central | MOH | IESS | Steering |
| CORUS | Public | Human health | Health authority | Public health | Central | MOH | IESS | Steering  Investigation of suspected cases |
| DPSP | Public | Human health | Health authority | Public health | Central | MOH | IESS | Coordination  Investigation of suspected cases |
| Health districts | Public | Human health | Health authority | Public health | Local | MOH | IESS | Coordination  Investigation of suspected cases |
| CSPS | Public | Human health | Health care institution | Human medicine | Local | MOH | IESS | Data collection |
| Hospitals | Public | Human health | Health care institution | Human medicine | Central | MOH | IESS | Data collection |
| CHR | Public | Human health | Health care institution | Human medicine | Local | MOH | IESS | Data collection |
| CHU | Public | Human health | Health care institution | Human medicine | Central | MOH | IESS | Data collection |
| Private doctors | Private | Human health | Private practitioners | Human medicine | Local | MOH | IESS | Data collection |
| ASBC | Public | Human health | Community Agent | Human medicine | Local | MOH | IESS | Data collection |

ASBC: community-based health worker; CHR: regional hospital centre; CHU: university hospital centre; CNGE: national committee for outbreak management; CORUS: health emergency response operations centre; CSPS: health and social promotion centre; DFRC: directorate of wildlife and hunting resources; DGSV: general directorate of veterinary services; DPEEVCC: provincial directorate of the environment, green economy and climate change; DPRAH: provincial directorate of animal resources and fisheries; DPSP: directorate of population health protection; DREEVCC: regional directorate of the environment, green economy and climate change; DRRAH: regional directorate of the ministry of animal resources and fisheries; DSA: directorate of animal health; LNE: national livestock laboratory; LRE regional livestock laboratory; MEEVCC: ministry of the environment, green economy and climate change; MRAH: ministry of animal resources and fisheries; MS: ministry of health; PF: forestry post; PV: veterinary post; RESUREP: epidemiological surveillance network for animal diseases; SSEF: wildlife surveillance system; SSEI: integrated epidemiological surveillance system; VVV: village volunteer extension workers; ZATE: technical support zone for livestock.
